# Simultaneous Encapsulation of Probiotic Bacteria (*Lactococcus lactis*, and *Lactiplantibacillus plantarum*) in Calcium Alginate Hydrogels

**DOI:** 10.3390/gels11010034

**Published:** 2025-01-03

**Authors:** Marko Vinceković, Luna Maslov Bandić, Fabijan Oštarić, Marta Kiš, Nevijo Zdolec, Ivan Marić, Suzana Šegota, Hana Zelić, Nataša Mikulec

**Affiliations:** 1 Department of Chemistry, Faculty of Agriculture, University of Zagreb, Svetošimunska 25, 10000 Zagreb, Croatia; lmaslov@agr.hr (L.M.B.); hana.zelic51@gmail.com (H.Z.); 2 Department of Dairy Science, Faculty of Agriculture, University of Zagreb, Svetošimunska 25, 10000 Zagreb, Croatia; fostaric@agr.hr (F.O.); nmikulec@agr.hr (N.M.); 3 Faculty of Veterinary Medicine, University of Zagreb, Heinzelova 55, 10000 Zagreb, Croatia; mkis@vef.unizg.hr (M.K.);; 4 Laboratory for Information Systems, Division of Electronics, Ruđer Bošković Institute, Bijenička c. 54, 10000 Zagreb, Croatia; imaric@irb.hr; 5 Laboratory for Biocolloids and Surface Chemistry, Ruđer Bošković Institute, Bijenička c. 54, 10000 Zagreb, Croatia; ssegota@irb.hr

**Keywords:** simultaneous encapsulation, *Lactococcus lactis*, *Lactiplantibacillus plantarum*, calcium alginate hydrogel

## Abstract

Encapsulation in alginate hydrogel microspheres is an effective method for protecting and improving the survival of lactic acid bacteria in different environments. This research aims to expand the knowledge about the structure/property relationship of calcium alginate microspheres loaded with a mixture of autochthonous probiotic bacteria (*Lactococcus lactis* and *Lactiplantibacillus plantarum*). A novel hydrogel formulation (FORMLAB) was prepared by ionic gelation and the molecular interactions between the FORMLAB constituents, surface morphology, structure, swelling degree, and release profile were characterized. The simultaneous encapsulation of two bacterial cultures in the same compartment does not diminish their viability. The binding of calcium ions to bacterial cells creates favorable conditions for the propagation of the encapsulated bacteria. The molecular interactions between the FORMLAB constituents are complex, involving mainly hydrogen bonds and electrostatic interactions. With a very high degree of swelling followed by low crosslinking, the surface of the microspheres covered with bacterial cells and diffusion through the hydrogel matrix allow for the delivery of probiotics at the right time. The findings suggest that bacterial cells are efficiently delivered from calcium alginate microspheres, offering promising applications in the development of functional foods, especially in cheese production.

## 1. Introduction

Probiotics, particularly *Lactococcus lactis* (*Lc. lactis*) and *Lactiplantibacillus plantarum* (*L. plantarum*), have received significant attention due to their ability to promote gut health, modulate the immune system, and prevent diseases [1,2]. Despite their potential health benefits, one of the main challenges in probiotic applications is ensuring the viability of bacterial strains during processing, storage, and in complex food matrices and the gastrointestinal tract [3,4]. Environmental factors such as extreme pH levels, temperature fluctuations, and exposure to bile salts can drastically reduce the effectiveness of probiotics before they reach their intended site. Therefore, it is very important to protect probiotics and ensure their stability in different physical environments.

Lactic acid bacteria are well known for their role as probiotics which, when consumed in appropriate amounts, provide health benefits to the host. The fundamental problem in the use of lactic acid bacteria is how to extend their shelf life and viability during storage, within complex food matrices, and during consumption. According to Jurić et al. [4], encapsulation is a practical and effective way to extend their survival and activity. Encapsulation is a physical, chemical, or mechanical procedure incorporating bioactive components within another substance. The ionic gelation technique is a standard encapsulation method due to its manufacturing simplicity, applicability, cheap cost, and ability to employ a range of biopolymers as encapsulating materials [5]. Among various encapsulation materials, alginate, a natural polysaccharide, is widely known for its biocompatibility, biodegradability, and ability to form gel-like microspheres with divalent cations such as calcium [6]. Alginate microspheres provide a protective matrix that shields the bacterial cells from environmental conditions and facilitates a controlled release of probiotics in complex food systems and the gastrointestinal tract [7]. The simultaneous encapsulation of multiple probiotic strains offers further advantages by enhancing the probiotic effect through synergistic interactions between the strains. Studies have shown that simultaneous encapsulation can improve bacterial survival under adverse gastrointestinal conditions and optimize their release, improving health outcomes [8,9]. The simultaneous encapsulation strategy has increased the stability and viability of probiotic cultures, making it an attractive approach for functional food development [10].

The manufacture of cheese from the milk of various dairy animals (cows, goats, sheep, and buffalos) is a traditional activity in Mediterranean countries [11]. The majority of Croatian autochthonous cheeses are semi-hard and hard cheeses made from raw full-fat ewe’s milk that ripen for at least 3 weeks. Paški sir is a well-known autochthonous Croatian cheese made solely on the island of Pag from the milk of the autochthonous Pag sheep. Raw and pasteurized milk, with or without additional dairy cultures, are used in the Pag cheese-making process. Similarly to other autochthonous types of cheese in Mediterranean countries, Paški sir spontaneously ferments due to naturally occurring lactic acid bacteria [12,13,14]. Lactic acid bacteria are Gram-positive bacteria that produce lactic acid from various carbohydrates during the fermentation process. The fermentation step performed by lactic acid bacteria is one of the most critical phases during cheese preparation and manufacture. During cheese fermentation and production, lactic acid bacteria cultures have several very important tasks including the production of lactose (small molecules of monosaccharides) from the milk’s polysaccharides, peptides and free amino acids from proteins, and fatty acids from lipids, having the most important role in the formation of the cheese’s flavor [15,16].

Recently, we have shown the great potential for maintaining authenticity in cheese production by using a microencapsulated form of lactic acid bacteria isolated from the abomasum of lambs and natural rennet in producing hard sheep cheese, “Paški sir” [17].

This work aims to expand knowledge about the relationship between the structure and properties of calcium alginate microspheres (ALG/Ca) with the simultaneously encapsulated mixture of bacteria *Lc. lactis* and *L. plantarum* (LAB) and to increase the possible applications, especially in fermented milk products. By investigating the molecular interactions between the constituents of the microsphere formulation (FORMLAB), microsphere surface morphology, and release profile, this research seeks to enhance the delivery system for probiotics. These findings could contribute to developing more effective probiotic supplements and functional foods with enhanced stability, viability, and therapeutic efficacy.

## 2. Results and Discussion

The results are presented and discussed in two sections. The first section analyzes the morphological properties of LAB and its interaction with calcium ions. The second section describes LAB survival in microspheres and essential FORMLAB physicochemical properties.

### 2.1. Interaction Between LAB and Calcium Ions

#### 2.1.1. Morphology and Size of LAB Cells

**Figure 1:**
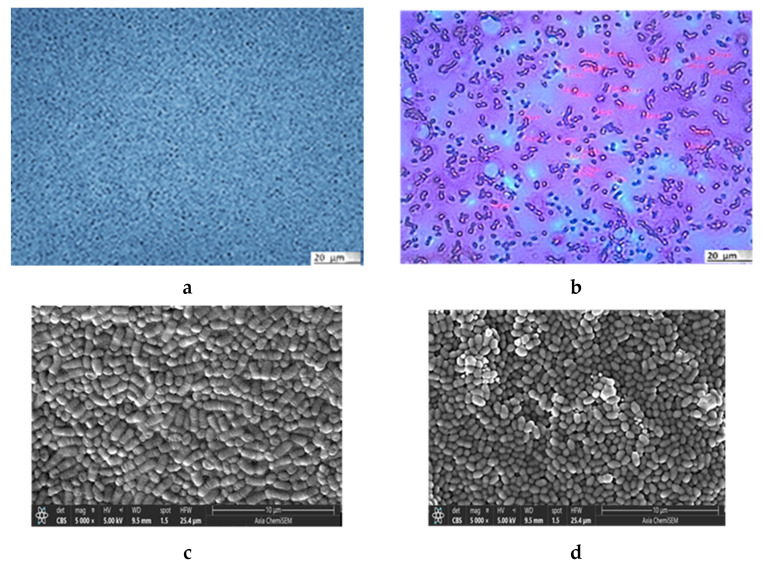

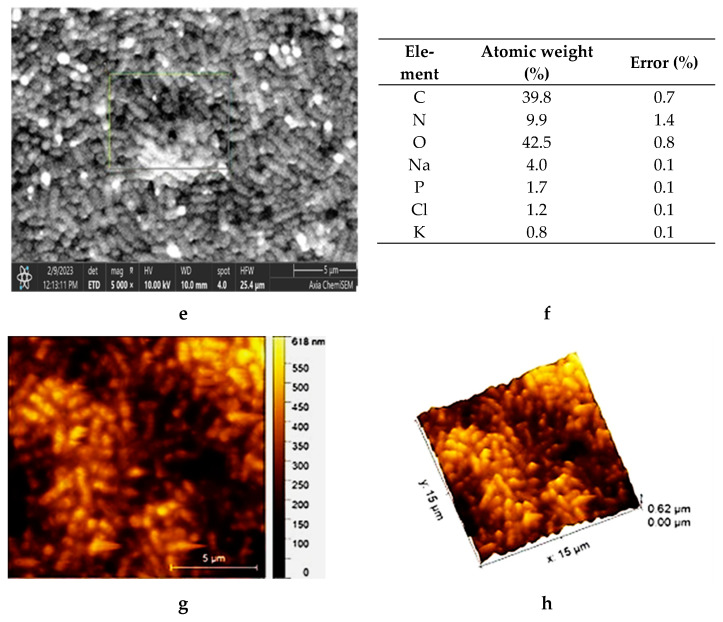
Microphotographs of *L. plantarum* and *Lc. lactis* cell mixture obtained with LM (**a**) without and (**b**) with staining. SEM images of (**c**) *L. plantarum* cells and (**d**) *Lc. lactis* cells. SEM microphotograph of *L. plantarum* and *Lc. lactis* cell mixture: (**e**) surface morphology and (**f**) surface elemental analysis using dispersive X-ray spectroscopy (expressed in the atomic weight percent). AFM of *L. plantarum* and *Lc. lactis* cell mixture: (**g**) detailed 2D topographic height image with dimensions of 15 × 15 µm^2^, and (**h**) 3D-AFM height image with dimensions of 5 × 5 µm^2^, respectively. Bars are indicated on each image.

Both microscopic analyses, light microscopy (LM) (Figure 1a,b) and scanning electron microscopy (SEM) (Figure 1c,d), clearly show the difference in the shapes of the two bacterial cells: the rod-like structures of *L. plantarum* cells and the almost round structures of *Lc. lactis* cells (Figure 1a). Staining with the Gram staining method better emphasized the differences in the shapes (Figure 1b). The SEM image of the *L. plantarum* cells (Figure 1c) revealed a rod-like shape with rounded ends (a width of about 0.9 to 1.2 μm and a length of 0.5 to 1.5 μm) and of the *Lc. lactis* cells (Figure 1d) revealed a round shape (with a width of about 0.7 to 1.0 μm, and a length of 1.0 to 8.0 μm). The measured values are consistent with data in the literature [18]. The SEM image of the bacterial mixture is shown in Figure 1e, and the results of the EDS analysis in Figure 1f indicate the presence of elements that build the cells of lactic acid bacteria [19].

The atomic force microscope (AFM) imaging of the LAB revealed a uniform surface of elongated oval-shaped cells (Figure 1g,h). The average length of the cells was about 1.4 µm, while the average width was about 0.57 µm, confirming their uniform size and shape distribution. In particular, the Feret dimension (the ratio of the maximum and minimum Feret diameters) analysis of the cells (Fd is about 0.43 µm) indicates morphologically uniform cells, as reported previously [20].

#### 2.1.2. Influence of Calcium Ions on Charge and Size of LAB Cells

The cell wall of lactobacillus bacteria is a dynamic structure that influences their survival, antimicrobial activity, and interactions with the world around them. It mainly contains multilayered peptidoglycan, teichoic acid, polysaccharides, and proteins [21]. Calcium ions are required for intracellular signaling and the regulation of numerous cellular processes such as cell division and development, motility, stress response, and host–pathogen interactions [22,23]. In addition, calcium ions are required for bacterial cell walls and teichoic acid stability [24]. The binding of calcium ions to cell wall functional groups can enhance the growth and activity of lactic acid bacteria.

**Figure 2:**
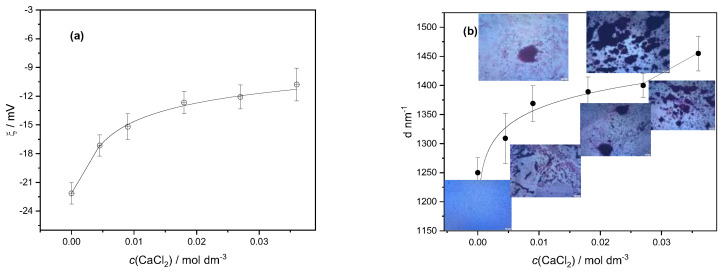
Variation in (**a**) average zeta potential (ζ) and (**b**) average hydrodynamic diameter (d) of particles with calcium chloride concentration (c(CaCl_2_)) of suspended mixtures of lactic acid bacteria cells. Standard deviations are denoted. Inserted microphotographs illustrate the growth of aggregates of bacteria with an increase in calcium ion concentration.

The effects of various calcium ion concentrations on the zeta potential and size of the LAB cells suspended in water are presented in Figure 2. The LAB cells were easily suspended in water, which indicated their hydrophilicity. They are negatively charged, having zeta potential ζ = −22.27 ± 0.47 mV. The electrical double layer between cells is known to be neutralized by calcium, and calcium is also known to stimulate specific adhesive contacts with protein and polysaccharide adhesin molecules on the cell surface [25]. As shown in Figure 2a, the bacterial cells become less negatively charged with an increasing concentration of calcium ions due to the electrostatic binding of calcium cations on the cell surface. Lowering the negative potential of the bacterial cells resulted in their accumulation in larger aggregates (Figure 2b). Even at the highest tested concentration of calcium chloride, there was no complete neutralization of the charge of the suspended LAB cells, which aligned with previous research [4]. It is known that LAB aggregation and adhesion to solid surfaces play a fundamental role in biotechnology, food processing, and cheese production [26].

### 2.2. Physicochemical Characterization and Analysis of Microspheres

#### 2.2.1. Identification of Molecular Interactions in Microspheres

**Figure 3:**
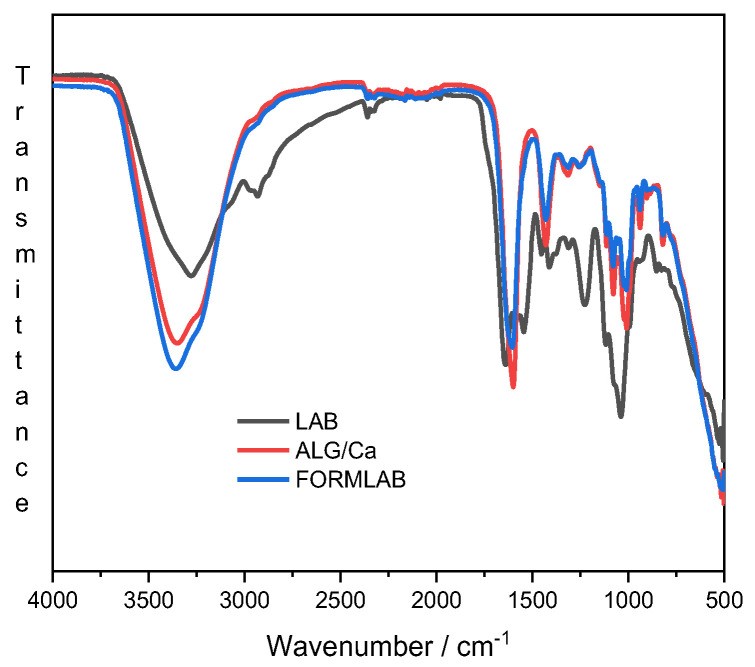
FTIR spectra of freeze-dried LAB (black line), ALG/Ca (red line), and FORMLAB (blue line) microspheres.

To gain insight into the complex molecular interactions in FORMLAB, FTIR analysis of single *Lc. lactis* or *L. plantaraum* bacteria cells and their mixture as well as of the prepared FORMLAB microspheres was performed. The FTIR spectra of *Lc. lactis* and *L. plantarum* almost overlap with the freeze-dried LAB spectrum and are not shown. Figure 3 presents only the spectra of their mixture, ALG/Ca and FORMLAB.

The spectrum of the LAB (black line) shows several characteristic peaks in certain wavenumber ranges that are characteristic of lactic acid bacteria: (i) fatty acids in the bacterial cell membrane (from 3000 to 2800 cm^−1^), (ii) amide bands of proteins and peptides (from 1800 to 1500 cm^−1^), (iii) mixed region: proteins and fatty acids (from 1500 to 1200 cm^−1^), (iv) polysaccharides within the cell wall (from 1200 to 900 cm^−1^), and (v) fingerprint region containing bands that cannot be assigned to specific functional groups (from 900 to 500 cm^−1^) [27]. The absence of LAB characteristic bands in the FORMLAB spectrum (blue line) confirms successful encapsulation.

The spectrum of FORMLAB exhibits characteristics of the calcium alginate spectrum (red line): a broad and strong peak, which corresponds to the stretching of -OH in the range of 3300 to 3500 cm^−1^; the bands at 1595 and 1405 cm^−1^ corresponding to the asymmetric and symmetric stretching peaks of carboxylate (COO^−^) groups; the C-O-C antisymmetric stretching attributed to bands in the 1081 to 1021 cm^−1^ area and 1081 to 1021 cm^−1^ region. Other vibrations were observed as a result of C-N, N-H, and O-H stretching in the 1190 to 920 cm^−1^ and 3200 to 3600 cm^−1^ regions, respectively.

The differences between the ALG/Ca and FORMLAB spectra are visible in the range of -OH stretching vibration bands and in the range of carboxylate ion stretching vibrations. More intense peaks and somewhat broader bands of the -OH stretching vibrations as well as the shifting and slightly lower peak intensity of carboxylate ion stretching vibrations indicate enhanced intermolecular hydrogen bonds and reduced electrostatic interactions in the FORMLAB microspheres, respectively. A decrease in the peak intensity at 1596 cm^−1^ and a narrower C-O-C stretching region indicate changes in the Ca^2+^ availability that affect the number of alginate strands held together in the three-dimensional network and thereby alter the strength of the gel structure [28]. Changes in the Ca^2+^ availability can be ascribed to the binding of calcium ions to the LAB cells and electrostatic repulsions between the biopolymer matrix and negatively charged bacterial cells. Similar changes in the location and strength of the bands of the FORMLAB microspheres compared with those of ALG/Ca were observed upon *Lactobacillus sakei* encapsulation into calcium alginate microspheres [4]. Changes in the FTIR spectra undoubtedly show structural differences in the microspheres prepared without and with LAB due to changes in the hydrogen bonding and electrostatic interactions.

#### 2.2.2. Morphology and Size of Microspheres

**Figure 4:**
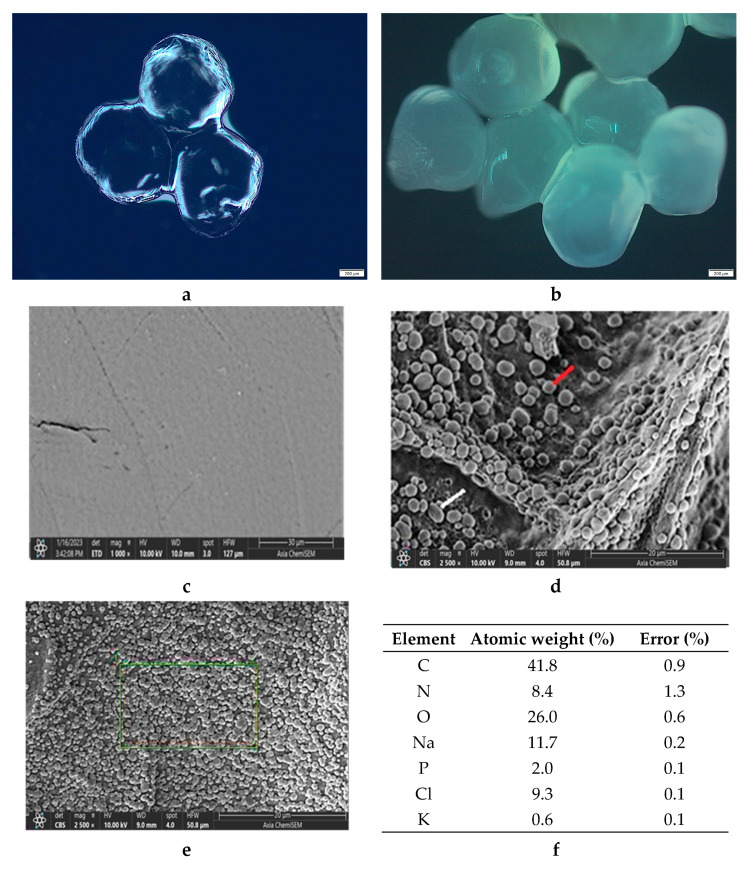
LM microphotographs of (**a**) ALG/Ca and (**b**) FORMLAB microspheres. SEM microphotograph of (**c**) ALG/Ca (*Lc. lactis* marked with the red line and *L. plantarum* with the white line) and (**d**,**e**) FORMLAB surface, and (**f**) surface elemental analysis using dispersive X-ray spectroscopy (expressed in the atomic weight percent). Bars are indicated on each image.

Microphotographs of the wet ALG/Ca and FORMLAB microspheres obtained with LM and the surface morphology obtained with SEM are shown in Figure 4. Both the ALG/Ca (Figure 4a) and FORMLAB (Figure 4b) show, under LM, a spherical egg shape, which could be due to the high surface tension of the crosslinking solution using calcium chloride [29]. The presence of LAB causes a change in the network structure of the calcium alginate gel, that is, the degree of crosslinking caused by molecular interactions between the ingredients decreases, which affects the morphology of the resulting microspheres. The size of the ALG/Ca microspheres is approximately 891 µm, and that of FORMLAB is approximately 1080 µm. Significant and obvious changes in the size, shape, and surface of both microspheres occur when they are freeze-dried to a constant mass. Both microspheres have size reductions of about 52% when moisture and water are lost. The SEM surface microphotographs of the ALG/Ca (Figure 4c) and FORMLAB (Figure 4d) significantly differ. The ALG/Ca surface is relatively smooth with numerous pores, while the FORMLAB surface is covered with cells of both bacteria (round *Lc. lactis* (marked with the red line) and rod-shaped *L. plantarum* (marked with the white line)). This is an important fact for a timely release process during cheese preparation and production [17]. The EDS spectra analysis of the area nearest to the FORMLAB surface (the electron probe can penetrate to a depth of about 1 μm) exhibited major surface elements corresponding to oxygen and carbon (Figure 4f). The detected nitrogen and phosphorus as well as potassium, sodium, and chlorine revealed the presence of characteristic LAB biomolecules (such as proteins, nucleic acids, and carbohydrates). The presence of bacteria on the surface of the calcium alginate microspheres was already observed for *Lactobacillus sakei* [4] and *Trichoderma viride* encapsulated in calcium alginate [30].

#### 2.2.3. The Survival of LAB Strain in FORMLAB

**Figure 5:**
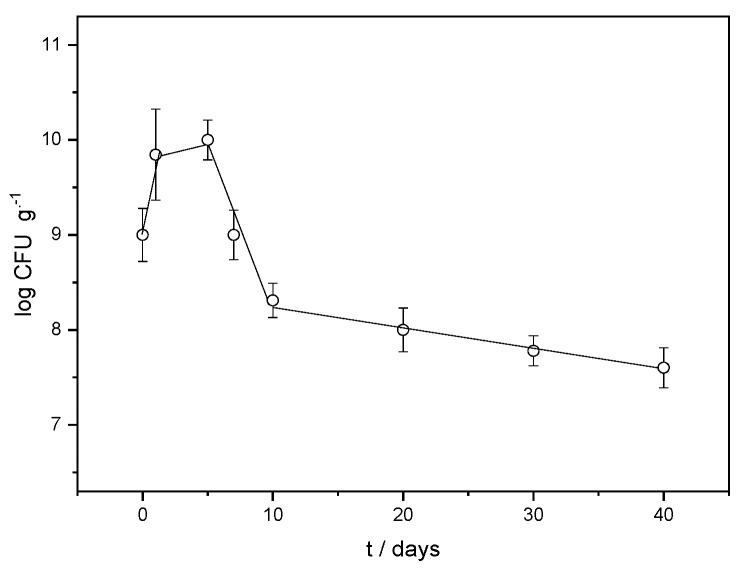
Variation in LAB strain abundance (log CFU g^−1^) in the FORMLAB microspheres with time (t).

The variation in the LAB strain abundance in the FORMLAB microspheres with time is presented in Figure 5. On the fifth day, it reaches the highest value, after which the concentration gradually decreases. In the study by Corbo et al. [31], the number of *L. plantarum* bacteria immediately after encapsulation was about 3.5 log CFU mL^−1^. The highest concentration of 6 log CFU mL^−1^ was also measured on the fifth day. The increased CFU value in FORMLAB can be explained by the positive influence of Ca^2+^ ions on the growth of LAB [32] as well as by the more equally dispersed bacterial cells after encapsulation (less clumped than before encapsulation) in the mixture to be plated [33]. The viable fraction exceeds the minimum 10^7^ viable bacteria per gram of product required by international standards for fermented products [4,34]. This indicates less need to add LAB to the fermentation matrix due to the high encapsulation yield, considering their numbers in the prepared solution. Namely, the original bacterial population employed for encapsulation was 9.0 ± 0.1 log CFU mL^−1^, and the viable fraction after entrapment was 9.85 ± 0.1 log CFU g^−1^. With suitable yields, the ionic gelation of alginate is a very promising option as a simple, efficient, and inexpensive process for various purposes [35]. Numerous studies of LAB encapsulation investigate the possibility of achieving high values of encapsulation yields, but the values presented vary greatly [5,36]. Different molecular interactions between the encapsulated LAB cells and other microsphere constituents can explain this.

We can conclude that the microspheres create favorable conditions for the propagation of the encapsulated strains by increasing their number. However, the influence of the BHI broth, a suitable medium for culture propagation, should not be ignored either. A similar result has already been observed in *Lactobacillus sakei* in microspheres, demonstrated by Jurić et al. [4].

#### 2.2.4. In Vitro LAB Release from FORMLAB Microspheres

The mechanism of LAB release gave us insight into the capacity of a gel network to retain cells within the microspheres. By suspending hydrophilic polymer microspheres in the medium, the medium penetrates through the surface of the microspheres, and the encapsulated agent is released by diffusion through the microspheres [37]. In contact with a solution, biopolymer chains are gradually broken down by hydrolysis, and the gel network of calcium alginate weakens until the degree of entanglement of the macromolecules is no longer sufficient to prevent significant swelling of the particles. Additionally, the degradation products create an osmotic pressure that continuously increases within the system and lures solution into the microspheres.

**Figure 6:**
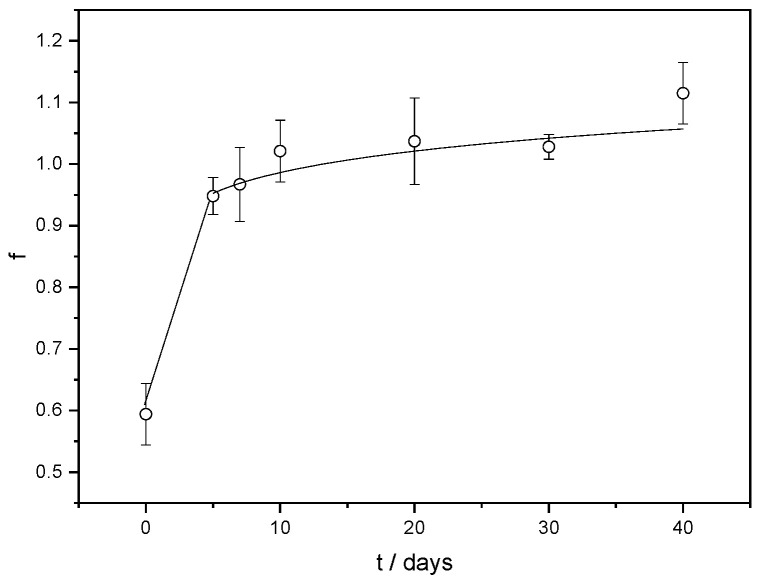
Fraction of LAB (f) release from FORMLAB microspheres with time (t). The data are shown as a mean value with standard deviation.

Crosslinks regulate how much hydrogels swell, and *S_w_* can be used to measure the extent of crosslinking [38]. Compared to ALG/Ca (*S_w_* ≅ 48%) generated under the same preparation conditions, the swelling degree of FORMLAB is nearly three times higher (about 155%). The increase in S_w_ can be explained by the effect of the encapsulated LAB cells on the crosslinking and weakening of the network structure due to mechanical interactions and the electrostatic repulsions between the negatively charged LAB strain and free parts of alginate chains (the zeta potential of the calcium alginate matrix is about −10 mV) [39]. A similar finding has already been observed with the encapsulation of *Trichoderma viride* [30] and *Lactobacillus sakei* [4]. A lower crosslinking density facilitates release, while a high crosslinking density slows down release. The release profile of the LAB from the FORMLAB microspheres is presented as a change in the released average number of cells (log CFU mL^−1^) in solution with time (Figure 6).

At the beginning of LAB release, the majority of the cells are rapidly released. This is consistent with microscopic observations showing the bacterial cells’ location on the surface of the FORMLAB microspheres (Figure 4d). The rapid release of a large amount of cells is a very important step in the cheese ripening process [4]. During further observation, the number of released LAB cells gradually increased. The Korsmeyer–Peppas model was used to determine the underlying mechanism controlling the rate of LAB release [40]. A simple empirical equation can discriminate distinct kinetics and mechanism of release according to this approach:

f = *k* t*^n^* (1)

where f is the fraction of the released LAB at time t, *k* is a kinetic constant characteristic for a particular system considering structural and geometrical aspects, and *n* is the release exponent, representing the release mechanism.

A release exponent value less than 0.43 (*n* = 0.1) indicates Fickian diffusion as a release rate-controlling mechanism [40]. The high water content in the swollen microspheres is beneficial for the diffusion of the encapsulated ingredients, ensuring the survival, migration, and proliferation of encapsulated cells [41].

**Figure 7:**
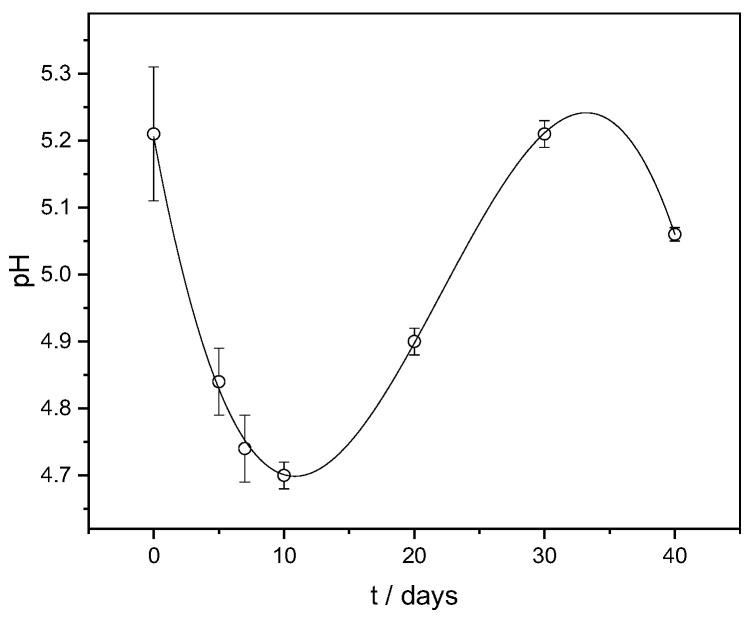
pH change with time (t) in a model system. The data are shown as mean values with standard deviation.

In parallel with the LAB release monitoring, the pH value change was also measured at the same time intervals. Lactic acid bacteria acidify water through lacto-fermentation during the breakdown of carbohydrates into lactic acid and carbon dioxide. The ability of the LAB released from the FORMLAB microspheres to acidify the model system was confirmed by the decrease in the pH value from the initial value of 5.21 to the lowest value of 4.70 over 10 days (Figure 7). This is consistent with research showing that the rapid acidification of fermented milk with microspheres containing encapsulated probiotic bacteria is an important first step in the cheese ripening process [17,42]. First, the pH decreases, and subsequently, the pH increases until 30 days, which is a consequence of complex physicochemical processes involved when hydrophilic biopolymer microspheres are suspended in a medium. The influence of the medium’s ingredients in which the microspheres are suspended is also important. The subsequent pH increase after 10 days can be explained by the ion exchange between the cations present in the release medium (BHI broth) and Ca^2+^ associated with the carboxyl groups of the alginate. The replacement of Ca^2+^ causes an increase in the distance between the alginate chains, which leads to the partial disintegration of the microspheres.

## 3. Conclusions

This study demonstrates the successful simultaneous encapsulation of a *Lc. Lactis* and *L. plantarum* mixture into calcium alginate microspheres. It is focused on understanding bacterial survival, the interaction with calcium ions, and the structure/property relationship of FORMLAB microspheres.

The simultaneous encapsulation effectively maintained the viability of the LAB strains for up to 40 days, with the highest concentration observed on the fifth day. The study also revealed the impact of calcium ions on the electrostatic properties of the LAB cells, which facilitated the formation of stable microspheres with high encapsulation yields.

LAB encapsulation altered the molecular interactions (intermolecular hydrogen bonds and electrostatic interactions) within the ALG/Ca gel. The addition of LAB changes the smooth texture of the ALG/Ca surface to a rough FORMLAB surface covered with cells of both bacteria. The degree of swelling of the FORMLAB microspheres is almost three times higher than that of the ALG/Ca microspheres, forming a less dense network structure.

The high water content of the swollen microspheres is suitable for the migration of encapsulated LAB through the FORMLAB matrix because Fickian diffusion was found to be the release control mechanism. The presence of LAB cells on the surface of the FORMLAB and the strong swelling is responsible for the rapid release of most of the cells into the medium, which was crucial for maintaining the desired pH levels necessary for cheese fermentation.

The study’s findings highlight the potential of FORMLAB microspheres as a promising delivery system for probiotics in functional foods, such as beverages and dairy products, particularly in cheese production, where they can enhance the stability and viability of the encapsulated strain.

## 4. Materials and Methods

### 4.1. Materials

#### 4.1.1. Chemicals

Sigma Aldrich (St. Louis, MO, USA) provided alginic acid sodium salt (CAS Number: 9005-38-3, M/G ratio: 1.56, molecular weight: 280,000 g mol^−1^). Kemika (Zagreb, Croatia) provided commercially accessible products CaCl_2_ and Na_3_C_6_H_5_O_7_ × 2H_2_O, and Merck (Darmstadt, Germany) delivered NaHCO_3_ and NaCl. All other compounds were of analytical quality and were utilized with no additional purification. 

#### 4.1.2. Bacterial Strain and Biomass Production

As previously reported, 150 isolates of lactic acid bacteria from raw sheep milk, lamb abomasum, cheese curds, and Pag island cheese were identified and characterized [11]. Based on the established biochemical properties, the *L. plantarum* strain from the lamb abomasum and the *Lc. lactis* strains from raw sheep milk were selected for encapsulation. To generate sufficient biomass for encapsulation, a pure culture of each strain was inoculated into 300 mL de Man, Rogosa, and Sharpe (MRS) broth (Merck Millipore, Burlington, MA, USA) and incubated at 30 °C for 24 h. The broth containing the propagated culture was then centrifuged at 3800 rpm for five minutes (Eppendorf 5804r, Hamburg, Germany), the supernatant was discarded, and cells from both broths were resuspended in 15 mL Brain Heart Infusion (BHI) broth (Thermo Fisher Scientific, Waltham, MA, USA). Before encapsulation, the number of cells in the prepared solution was determined according to the method of Kiš et al. [17].

#### 4.1.3. Microsphere Preparation

According to Vinceković et al. [43], the microsphere formulation was made in a single step by alginate ionic gelation at room temperature in a sterile environment. Briefly, 15 mL of LAB cell mixture was added to 85 mL of sodium alginate solution, and the mixture was lightly mixed for 10 min (Biosan Orbital Shaker-Incubator ES-20, Riga, Latvia) before being homogenized. The final concentration of sodium alginate solution was adjusted to 1.5% *w*/*v*. At 600 Hz vibration frequency and 121 mBar pressure, the mixture was dripped into 100 mL of a CaCl_2_ solution (1% *w*/*v*) through an encapsulator nozzle size of 750 µm (Encapsulator Büchi-B390, Bütchi Labortechnik, Flawil, AG, Switzerland). The prepared microspheres were maintained at room temperature for an additional 30 min to encourage gel strengthening. The microspheres were cleaned three times with sterile distilled water to eliminate the residue of CaCl_2_, filtered through a Büchner funnel, and kept at 4 °C until further research. Aseptic conditions were ensured throughout the whole process.

### 4.2. Methods

#### 4.2.1. Determination of Zeta Potential and Size of Bacterial Cells in Suspension

The electrostatic charge and zeta potential (ζ) of LAB cell mixtures suspended in water and calcium chloride solutions, as previously described [4], were determined using the Zetasizer Ultra (Malvern Panalytical, London, UK) equipped with a 632.8 nm He-Ne laser, using the Multi-Angle Dynamic Light Scattering (MADLS^®^) technology.

The hydrodynamic diameter (d) was estimated using the Einstein–Stokes equation, which assumed a spherical aggregate. Results are presented as the mean value of at least three to six measurements.

#### 4.2.2. Microscopic Observations

*Lc. lactis* and *L. plantarum* cells and their mixture as well as ALG/Ca and FORMLAB were analyzed by microscopic techniques: (i) light microscopy (LM) (Leica MZ16a stereomicroscope, Leica Microsystems Ltd., Balgach, Switzerland)), (ii) scanning electron microscopy (SEM) (FESEM, model JSM-7000 F, Jeol Ltd., Akishima, Japan), and (iii) atomic force microscopy (AFM) (Nanosurf CoreAFM, Nanosurf AG, Liestal, Switzerland)).

LM was used to measure the average diameter of bacterial cells and microspheres (wet and dry) using Olympus Soft Imaging Solutions GmbH, version E_LCmicro_09Oct2009. Forty microspheres were chosen randomly from batches generated in triplicate to determine the size distribution, as previously described [4].

The samples for SEM analysis were placed on high-conductive graphite tape. FESEM was linked to an EDS/INCA 350 (energy dispersive X-ray analyzer) manufactured by Oxford Instruments Ltd. (Oxon, UK). The ImageJ software (Phyris software 9.1.0.0198) was used to determine the size of bacterial cells and microspheres.

The AFM image was taken in WaveMode off-resonance imaging mode using CleanDrive photothermal excitation. The active vibration isolation table was built in the AFM xy stage, and the AFM was placed in our Acoustic Enclosure AE 350 (bottom left). The cantilever type was WM0.6AuD. This small cantilever is designed for operation with photothermal excitation. It has a detector side reflective gold coating, a resonance frequency of 350 kHz, and a spring constant of 0.6 N/m (nom.). The imaging speed was 2.5 Hz at an image resolution of 500 × 500 px.

#### 4.2.3. Fourier Transform Infrared Spectroscopy Analysis

The Fourier transform infrared spectroscopy (FTIR) spectra were recorded with the FTIR Instrument—Cary 660 FTIR (MIR system) spectrometer (Agilent Technologies, Santa Clara, CA, USA). Freeze-dried samples were mixed with potassium bromide to obtain pellets as previously described [4]. Spectral scanning was performed in the range of 400–4000 cm^−1^.

#### 4.2.4. Encapsulation Yield of LAB Cells in FORMLAB and Swelling Degree Determination

Detailed procedures for the determination of encapsulation yield (EY) and swelling degree (Sw) have been previously described [17,43]. The encapsulation yield was expressed as a percentage of viable cells used for the encapsulation and calculated as follows:

EY = (N_microspheres_/N_suspension_) × 100 (2)

where N_microspheres_ (in CFU per gram of microspheres) and N_suspension_ (in CFU per gram of solution) denote viable counts in alginate microspheres and cell suspension, respectively. *S_w_* of microspheres was calculated using the following equation:

*S_w_* = ((w − w_o_)/w_o_), (3)

where w_t_ is the weight of the swollen microspheres and w_o_ is their initial weight.

#### 4.2.5. The Survival and In Vitro LAB Cell Release from FORMLAB

The survival and release of LAB cells from FORMLAB microspheres were monitored for 40 days at room temperature in the same model system. A mass of 1 g of FORMLAB was sterilely weighed into Falcon tubes to which 10 mL of BHI broth was added. Two samples were taken on days 0, 5, 7, 10, 20, 30, and 40. After sampling, microspheres were separated from the medium, including controls. A mass of 1 g of microspheres separated from the BHI medium was dissolved in 9 mL of sterile sodium bicarbonate/trisodium citrate solution (0.2 mol dm^−3^ NaHCO_3_, 0.06 mol dm^−3^ and Na_3_C_6_H_5_O_7_ × 2 H_2_O). The number of released cells was determined in the medium after microsphere separation. The number of cells in the microspheres and released in the medium was determined by decimal serial dilution in sterile saline solution (0.85% NaCl, Merck, Darmstadt, Germany). The selected dilutions were plated on MRS agar (Biolife, Milan, Lombardy, Italy) plates and incubated anaerobically at 30 °C for 48 h. The survival of the LAB strain in the model system was monitored by the number of bacteria using an automatic colony counter (Scan 1200, Interscience, Cantal, France) according to the method of Kiš et al. [17]. The fraction of released LAB (f) was calculated using the following equation:

f = (R_t_/R_tot_), (4)

R_t_ is the amount of LAB released at time t, and R_tot_ is the total amount of LAB loaded in FORMLAB.

#### 4.2.6. pH Measurements

The pH of BHI broth containing FORMLAB was measured before sampling at specific time intervals after suspension. At the same time, the pH of the BHI broth inoculated with free LAB cells was measured as a control. The pH of all samples was determined using a pH meter model BT-600 (Boeco, Hamburg, Germany).

#### 4.2.7. Data Analysis

Microsphere characterization experiments were carried out at room temperature in triplicate. The obtained data were analyzed with Microsoft Excel 2016 and XLSTAT statistical software (version 2021.1) add-in and IBM SPSS Statistics 22. Measurements were performed at room temperature in triplicate. All data were shown as a mean value with standard deviation. The pH and viable count of released cells and cells in microspheres were carried out in three independent experiments (n = 3). Two-way ANOVA was used to test for significant differences in pH and release and survival of LAB in microspheres.

## Data Availability

The original contributions presented in the study are included in the article. Further inquiries can be directed to the corresponding author.

## Abbreviations

ALG/Ca, calcium alginate microspheres; LAB, mixture of *Lactococcus lactis* and *Lactiplantibacillus plantarum*; LM, light microscope; SEM, scanning electron microscope; AFM, atomic force microscope; Fd, Feret diameter; CFU, the number of colony-forming units.
